# Sexual Seroadaptation: Lessons for Prevention and Sex Research from a Cohort of HIV-Positive Men Who Have Sex with Men

**DOI:** 10.1371/journal.pone.0008831

**Published:** 2010-01-21

**Authors:** J. Jeff McConnell, Larry Bragg, Stephen Shiboski, Robert M. Grant

**Affiliations:** 1 Gladstone Institute of Virology and Immunology, San Francisco, California, United States of America; 2 Department of Epidemiology and Biostatistics, University of California San Francisco, San Francisco, California, United States of America; 3 Department of Medicine, University of California San Francisco, San Francisco, California, United States of America; University of Sao Paulo, Brazil

## Abstract

**Background:**

Surveillance data on sexually transmitted infections (STIs) and behavioral characteristics identified in studies of the risk of seroconversion are often used as to track sexual behaviors that spread HIV. However, such analyses can be confounded by “seroadaptation”—the restriction of unprotected anal intercourse (UAI), especially unprotected insertive UAI, to seroconcordant partnerships.

**Methods:**

We utilized sexual network methodology and repeated-measures statistics to test the hypothesis that seroadaptive strategies reduce the risk of HIV transmission despite numerous partnerships and frequent UAI.

**Principal Findings:**

In a prospective cohort study of HIV superinfection including 168 HIV-positive men who have sex with men (MSM), we found extensive seroadaptation. UAI was 15.5 times more likely to occur with a positive partner than a negative one (95% confidence interval [CI], 9.1–26.4). Receptive UAI was 4.3 times more likely in seroconcordant partnerships than with negative partners (95% CI, 2.8–6.6), but insertive UAI was 13.6 times more likely with positives (95% CI, 7.2–25.6). Our estimates suggest that seroadaptation reduced HIV transmissions by 98%.

**Conclusion:**

Potentially effective HIV prevention strategies, such as seroadaptation, have evolved in communities of MSM before they have been recognized in research or discussed in the public health forum. Thus, to be informative, studies of HIV risk must be designed to assess seroadaptive behaviors rather than be limited to individual characteristics, unprotected intercourse, and numbers of partners. STI surveillance is not an effective indicator of trends in HIV incidence where there are strong patterns of seroadaptation.

## Introduction

Surveillance data on sexually transmitted infections (STIs) and results from behavioral studies of the risk of seroconversion have frequently been used as surrogate markers of trends in sexual behaviors that spread new HIV infections and signal changing trends in the HIV epidemic. The incidence of rectal gonorrhea among men who have sex with men (MSM) dropped precipitously with the onset of the AIDS epidemic in the 1980s. STIs, such as gonorrhea, usually become symptomatic soon after infection, and testing and treatment were simple and widely available, making STI surveillance an accessible indicator of HIV risk. At the same time, the rates of high-risk sexual behaviors and the incidence of HIV decreased [1]–[4]. Behavioral surveillance focusing on risk of seroconversion is relatively inexpensive and can be implemented with a wide variety of sampling strategies that can lead to findings that can be generalized. The concomitant decreases in the incidence of rectal gonorrhea, rates of high-risk sexual practices, and HIV incidence in the 1980s in San Francisco suggested that the former two measures were good surrogate indicators of potential trends in HIV incidence.

By the mid-1990s, behavioral studies revealed increasing rates of sex unprotected by condoms among MSM, and STIs rose accordingly [5], [6]. For example, from 1993 to 1999 in San Francisco, the proportion of MSM reporting multiple partners and unprotected intercourse increased while those reporting always using condoms dropped, and the rates of rectal gonorrhea rose [7]. However, more direct measures of HIV incidence in San Francisco did not show a concurrent increase [6]–[10]. This apparent discrepancy may have reflected changes in the infectivity of HIV due to widespread treatment [11] or other factors that specifically inhibited new HIV infections independently of other STIs.

However, changes in sexual mixing based on HIV-1 status could also partially explain discrepancies between trends in HIV and other STIs. Sexual behaviors adapted to the risk of HIV developed quickly among MSM in San Francisco, including abstinence, reduction in numbers of partners, avoiding anal sex, and condom use, and was credited with dramatic reductions in HIV incidence density before the end of the 1980s [1], [2].

The HIV epidemic has engendered an unprecedented sexual behavior surveillance infrastructure; a likewise unprecedented and large body of research on specific sexual practices, especially unprotected anal intercourse (UAI) among MSM has resulted. However, research that narrowly trained its sights on practices that could spread the epidemic was poorly suited to measure the role of behavior change in inhibiting the spread of the epidemic [12] ^(p71)^. Special adaptive strategies might be particularly relevant in communities in which the members correctly perceive themselves to be at risk of acquiring or transmitting infection. Newer trends in behaviors, discussed by French activists and intellectuals under the term “seroadaptation,” might reverse trends toward fewer partners and more condom use without concomitant increases in HIV incidence [13].

Data from a population-based sample of MSM living in California in 2002 led researchers in 2006 to conclude that knowledge of sexual partner's serostatus was associated with sexual practices frequently indicating an apparent seroadaptive strategy to reduce the risk of transmission without necessarily limiting numbers of partnerships or ruling out UAI. Serosorting, generally understood to refer to the selection of only concordant-serostatus partners, at least for UAI, was only one tactic characteristic of this seroadaptive strategy. While MSM often chose partners of discordant or unknown status those partnerships were less likely to involve anal intercourse or more likely to include condom use [14].

First described as a harm-reduction strategy in an Australian study, “strategic positioning” was another seroadaptive tactic in which an HIV-positive individual assumed the receptive role during UAI with a negative or unknown-status partner [15]. Because the risk of transmission from a receptive to an insertive partner during unprotected anal intercourse is minimal compared to the obverse [16]–[18], strategic positioning was another adaptive behavior that could mediate HIV incidence.

Other evidence of seroadaptation had been documented before the cohort study reported here although not in these terms. Limited serosorting was reported for Austrailian MSM between 1986 and 1991 [19]. In 1991–1992, 9% of a sample of HIV-positive MSM in Los Angeles reported insertive UAI and were more likely to do so with HIV-positive partners (odds ratio, 3.27) [20]. During the same period in the United Kingdom, one study found that in 15% of partnerships the serostatus of both partners was known to the respondent. Among this group, HIV-positive MSM were more likely to have UAI with HIV-positive MSM and less so with HIV-negative MSM (odds ratio, 1.64 vs. 0.24, p<.05) than with unknown-status partners [21]. During the latter half of the 1990s, patterns of serosorting and strategic positioning were evident in HIV-positive MSM in San Francisco and New York, even though the samples excluded the most successful seroadapters—those who reported no unprotected intercourse with negative or unknown-status partners [22]. In 1992, Hoff et al. found UAI much more likely to occur in seropositive concordant rather than discordant relationships (54% vs. 17%) among MSM in Portland, Oregon, and Tucson [23]. These examples are not an exhaustive list.

However, the present study is the first in which the data collection methodologies and analytic frameworks were chosen for the specific purpose of learning the degree to which seroadaptive tactics arose among sexual partnerships defined as at high-risk for HIV-transmission (involving HIV-positive MSM who practice UAI) that eluded both public health-sanctioned messages and empirical detection. It is also a unique effort to assess the potential impact of such tactics on decreasing exposures that might lead to transmission of new HIV infections. Whether this HIV prevention strategy arose from “grassroots” origins and, especially if they are effective, a theoretical question is raised: Do such tactics add up to a community-based prevention strategy? And under what conditions can they appear? Answers to these questions have bearing on the development of HIV prevention policy and programs.

As early as the mid-1980s, epidemiologists were using sexual mixing–based models of the epidemic that drew heavily on the theory and methods of social network research [24]–[29]. Social network studies have three fundamental characteristics: relationships rather than individuals are studied, relationships between individuals can be drawn or graphed in “sociograms” that illustrate social structure, and social structure can therefore be subjected to mathematical analysis and modeling. An advantage of these methods in studies of HIV seroincidence or superinfection is that exposure data are collected partner by partner, and the characteristics of each partner are documented separately. Thus, one can determine if partnership characteristics predict sexual risk and compare different analytic approaches to see how well they represent sexual risk in the sample overall.

## Methods

### Objectives

We had two primary objectives in this analysis. The first was to examine sexual partnership data for evidence of seroadaptive tactics among HIV-positive MSM in San Francisco before the recognition and discussion of serosorting in the peer-reviewed scientific literature, among public health officials, and in HIV-prevention policy. We tested the hypothesis that HIV-positive MSM reduced the risk of infecting partners both by serosorting and by strategic positioning—the restriction of UAI, especially unprotected insertive UAI, mainly to seroconcordant partnerships.

Our second objective was to provide an empirically based estimate of the impact of seroadaptation, especially serosorting and strategic positioning, on containing the HIV epidemic. In addition, we examined whether different methodological and analytic approaches to the study of HIV transmission risk could distort the impact of behavior changes like seroadaptive tactics on transmission incidence. Abstinence, avoiding anal intercourse altogether, always using condoms for intercourse, and other seroadaptive strategies were not examined empirically.

### Participants

Positive Partners was a prospective study of HIV-1 superinfection among HIV-positive men and women in San Francisco who reported frequent unprotected intercourse (>10 episodes) with at least one HIV-infected partner over the previous year. This eligibility criterion was optimized to look for evidence of HIV superinfection; hence, individuals with only negative or unknown-status partners were excluded. Prospective participants were screened for eligibility when they called the study in response to professional or personal referrals, brochures and fliers placed in gay and AIDS services venues, or advertisements printed bi-weekly in a gay-oriented newspaper. For the majority of participants (85.1%), a current HIV-positive sexual partner was also screened and enrolled. The enrollment of current seroconcordant partnerships was key to the superinfection study aims so that sexual exposure to a genetically divergent virus in an enrolled partner could be distinguished from exposure to unknown strains from other HIV-positive partners. Exposure to HIV-negative partners that could not lead to superinfection—or to unknown-status partners where the risk of exposure could only be estimated by HIV prevalence in the population—could be measured and controlled in estimating the risk of superinfection.

The data analyzed in this study were obtained from a subsample of participants in the Positive Partners Study consisting of all 168 MSM enrolled from January 2002 to December 2004 in San Francisco. We focused on this timeframe because subjects were observed during a period when seroadaptation had not been recognized or discussed in the scientific, public health, or popular literatures.

### Procedures

Behavioral interviews were conducted and biological specimens (blood and semen) were collected at enrollment and at the 1-year follow-up. The sexual partnerships and contacts of each participant during the 3 months before the intake and exit interviews (past 3 months) were documented. Sexual contacts were documented with a novel instrument based on egocentric social network methods [30]–[43], which were adapted to study sexual networks [44]–[56]. Egocentric social/sexual network designs depend on informants (egos) to characterize their partners (alters) and the relationships between them. To sample partnerships, we first selected all sexual partners in the past 3 months. During the self-administered interview module, participants were asked to fill out a “partner journal” in which each partner was described in a separate section, including a distinguishing identifier (first name, nickname, or other descriptor). Information collected on each partner in the past 3 months included basic demographics, HIV status, specific types and numbers of sexual contacts, and an indicator of partnership concurrency (during which month or months had sexual contact occurred). During the interviewer-administered module, we selected as many as four of the most recent of those partners (if four or more had been reported) plus the enrollment partner, if one existed, into a subsample for extensive characterization. An additional 29 questions solicited information on the characteristics of each subsample partner, their relationship, and the timing of sexual contacts. Our analysis indicated that the subsample partnerships were representative of the overall sample of partnerships in the past 3 months.

### Ethics

All subjects gave written informed consent to participate in the study. The protocol and consent forms were approved by the Committee on Human Research at the University of California, San Francisco.

### Analytic and Statistical Methods

**Figure 1:**
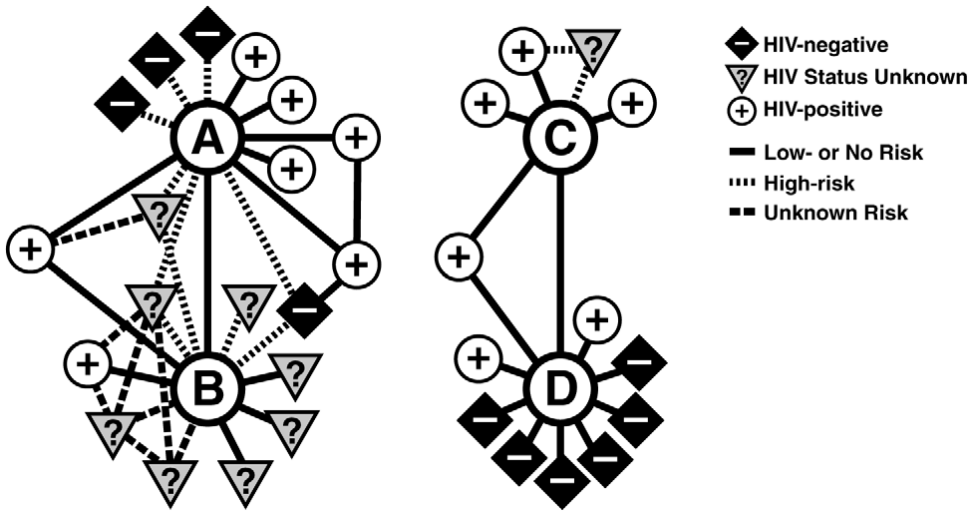
A network “sexiogram” differentiates sexual linkages that may or may not have caused new infections. The individuals represented by the labels A–B and C–D were couples enrolled in the study. The other nodes are sexual partners described by the enrollees. The partnerships were not necessarily concurrent and included all those reported during the 3 months before A's enrollment and those of D, who enrolled 6 months later. Thus, the partnerships spanned a 9-month interval altogether. In some analyses, all partnerships of these HIV-positive individuals known to practice UAI would be counted as potential transmission linkages. This diagram illustrates the preponderance of low-risk partnerships (solid lines) compared with potential transmission linkages (broken lines).

**Seroadaptation in Partnerships: A network “sexiogram” differentiated sexual linkages that may or may not have caused new infections.** In contrast to individual-based analysis, inclusion of partnership-specific information provides evidence of seroadaptation that may decrease exposures that could result in new HIV infections in partnerships. Partnership information also provides better resolution for evaluating the burden of exposure in the population. Figure 1 shows a reconstruction of partial sexual networks of two couples during the 3 months before enrollment in the Positive Partner's study, including all partners of each individual and diagrams of the connections with shared partners. This sexual network diagram illustrates variation in the risk of HIV transmission even among couples who practice UAI and have multiple serodiscordant partnerships. Individuals A and D were both HIV-infected, had multiple partnerships, frequently practiced UAI, and had HIV-negative partners. Many analyses would characterize them as equally likely to spread the epidemic. However, unprotected intercourse between seropositive partners does not pose a threat of new HIV infections. Therefore, D (a “complete” seroadapter) has not had UAI with any of his negative partners, and so we do not count any of his partnerships as likely transmission linkages. In contrast, while A is a “partial” seroadapter, we can count potential transmission linkages separately from low-risk partnerships. For this reason, sexual network data are particularly well suited to estimating the epidemiological impact of seroadaptation.

This diagram illustrates the key outcome variables, including whether unprotected receptive or insertive intercourse occurred in each partnership or whether sexual contact was limited to lower risk exposures such as protected anal intercourse, oral intercourse, or digital-genital/anal stimulation. The key predictor variables are whether the partner was known to be HIV-positive, negative, or was of unknown HIV status.

**Patterns of Partner Selection: Statistical Analysis.** Data were analyzed by alternate analytic strategies. Comparing different analytic approaches can help us evaluate whether they elucidate (or obscure) the discrepancy between increasing prevalence of behavioral risk and stable HIV incidence in San Francisco during the 1990s.

First, we used responses from the 168 participants as the unit of analysis, with information on linked partnerships limited to an aggregate summary from the respondent's perspective (e.g., number of known HIV-positive partners in the past 3 months). A self aggregated measure of sexual exposure was the standard approach to HIV behavioral risk assessment during the first 20 years of the epidemic although in the first 10–15 years partners status was rarely collected as it was here. Aggregated measures had the advantage that responses could be viewed as independent, and complex partnership histories that were summarized in a simplified form were amenable to standard methods of analysis. However, this approach did not capture key information that characterizes links between specific partnerships.

Second, we looked at the same data with all sexual partnerships reported by each respondent as the unit of analysis. Such “dyadic” data allow inferences about factors that distinguish different types of partnerships (e.g., primary versus casual or exchange-based relationships). Outcomes considered for these analyses include binary categorization of each partnership as reporting a particular practice (e.g., UAI) and the number of reported UAI episodes within each partnership. Binary outcomes were analyzed using logistic regression methods. Partnership-specific counts of acts reported over the 3-month period were modeled by Poisson regression, with the partner's HIV serostatus as the primary predictor. Separate models were fitted for different types of acts. Because individual participants could report about multiple partnerships, we would expect that resulting outcomes would not be independent of each other. Failure to control for this within-individual correlation in analyses could result in biased estimates of variability and incorrect inferences about the estimated effects in regression models. For this reason, all models were fitted using Generalized Estimating Equations (GEE) methods [57], and associated inferences were based on robust variance estimates accounting for such correlations.

## Results

### Study Cohort

From January 2002 to December 2004, 266 individuals contacted the study and were screened for eligibility in telephone interviews. Fifty-five were deemed ineligible because they reported insufficient unprotected intercourse exposure with a partner or because they and their partner were a transmission pair according to self-reported infection history. Of 211 individuals eligible for the study, 26 were not enrolled because they or their partner verbally declined to participate or did not keep enrollment appointments despite repeated contacts. Of 185 who completed the enrollment interview, four heterosexual couples were excluded from the analysis (the hypotheses tested here only apply to MSM) along with one individual who tested negative for HIV at enrollment. Eight of the remaining 176 MSM could not be included in the analysis because they declined to provide information on their sexual partnerships.

Demographically, the remaining sample of 168 MSM approximately reflected the epidemic in San Francisco from its beginning to 2004. The average age of participants was 41.3 years. The median individual income was $23,000. By race and ethnicity, 65.5% were white, 16.1% were African-American, 8.9% were Latino, and 9.6% were of other or mixed racial/ethnic background. By education, 15.5% had a high school diploma or less education, 23.8% were college graduates, and 21.4% had advanced degrees. Participants had first tested positive 1.5 weeks to 21 years before enrollment (mean ±SD, 9.8±5.8 years). At enrollment, the mean CD4 count was 502±276 cps/ml (range, 30–1,815), and the mean lowest reported CD4 count during the course of infection was 244±188 (range, 0–1,038).

The 168 participants reported a total of 1,059 sexual partnerships in the past 3 months involving 5,445 acts of intercourse. Some participants reported having sex only with a primary partner (29.2%), but the median number of partners was three, with a median of one act of UAI per partner.

### Comparative Analytic Strategy: Individual versus Partnership-Based Analysis, Elaboration by Frequency of Intercourse

First, we examined the data by using the individual respondent as the unit of analysis. To simulate an analysis in which only *individual-based* risk data were available, we aggregated partnerships for each informant. Then, to determined if the partnerships involved high-risk exposures, we categorized partners as HIV-positive, HIV-negative, or unknown status, and assessed whether UAI had occurred with each partner category during the 3-month period.

All 168 respondents knew themselves to be HIV-positive MSM who reported UAI. Therefore, in an individual-based analysis, they would be categorized among the highest-risk group for the transmission of HIV and spread of the epidemic: 57.1% had HIV-negative or unknown-status partners, 38.1% had UAI with HIV-negative or unknown-status partners, and 26.2% had insertive UAI with an HIV-negative or unknown-status partner. The group reported a mean of 4.67 partners in the past 3 months.

With the sexual exposure data aggregated in this fashion, efforts by participants to reduce the risk of new infections through seroadaptive choices, if any, were not apparent.

### Patterns of Seroadaptive Strategies Appear with Partnership as the Unit of Analysis

Using information from epidemiological studies, we prepared a *partnership-based* analysis of seroadaptive practices, including avoiding anal intercourse, serosorting, and strategic positioning. Partnerships were first categorized as serodiscordant (HIV-negative or unknown-status partners) or seroconcordant and then subcharacterized by sexual practices that involve different efficiencies of HIV transmission. For example, HIV-1 is more easily transmitted from an insertive positive partner than from a receptive positive partner. HIV-1 is more readily transmitted by anal intercourse than by oral-genital or hand-genital contact. Condom use is protective [16], [58].

**Figure 2:**
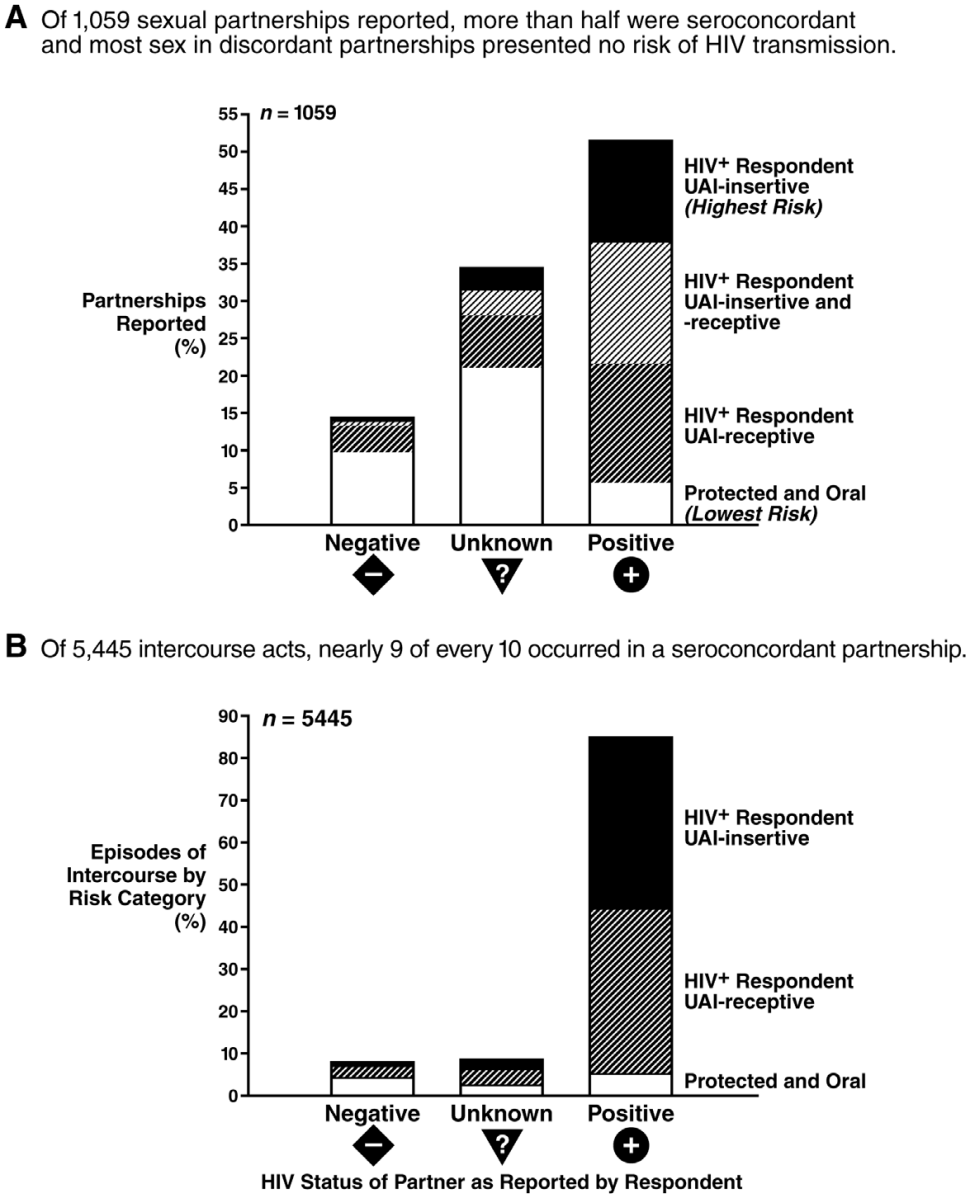
Sexual behavior by risk of HIV transmission and serostatus of partner. Sexual partnerships and episodes of intercourse reported by 168 seropositive individuals during the last 3 months reveal strong patterns of seroadaptation: Partnerships involving unprotected intercourse are predominantly seroconcordant, and 88.6% of all unprotected intercourse occurred with seropositive partners.

We examined the partner status and type of exposure for the 1,059 partnerships reported by these 168 individuals over the past 3 months (Figure 2a). Despite being ostensibly high-risk *individuals*, fewer than 1 in 5 reported partnerships that were serodiscordant (14.3%), the partner's HIV status was unknown in 34.4% of partnerships, and seroconcordant unions made up 51.4% of all reported partnerships.

Overall, a strong preference was apparent for intercourse without condoms. However, while 28.3% of the potentially serodiscordant partnerships involved UAI (191 of 674 UAI partnerships); the majority involved sexual practices with a lower risk or no risk for HIV transmission, including intercourse with condoms, oral sex, and hand/genital contact. Since the prevalence of HIV among MSM in San Francisco was estimated as 27.3% [59], perhaps one-quarter of unknown-status partners would actually constitute seroconcordant partnerships. As a result approximately 22.7% of UAI partnerships (those reported as negative or unknown status) actually presented a risk of a new infection. In contrast, when the possibility of causing a new infection was not a concern, practices were quite different: the majority (88.8%) of seroconcordant partnerships involved UAI.

Most (71.7%) UAI-partnerships were seroconcordant. The general tendency to select seroconcordant partners and, more specifically, for those partnerships to constitute the majority of UAI partnerships provided evidence of a pattern of seroadaptation that was not appreciated in the peer-reviewed literature before 2004 and has so far received only modest albeit growing attention [60]–[66].

We also found that strategic positioning had been adopted as part of the seroadaptive strategy, and it presumably further reduced the risk of HIV transmission from this sample. When UAI occurred with HIV-negative individuals, the positive partner was usually (76.0%) only a receptive partner. In only 12 (7.9%) partnerships with negatives was the positive partner insertive; in contrast, our informants were receptive in 62.5% and insertive in 57.9% of their seroconcordant UAI partnerships. These patterns indicate that assuming the receptive role was a harm-reduction strategy with discordant partners rather than a consistent preference among participants.

**Figure 3:**
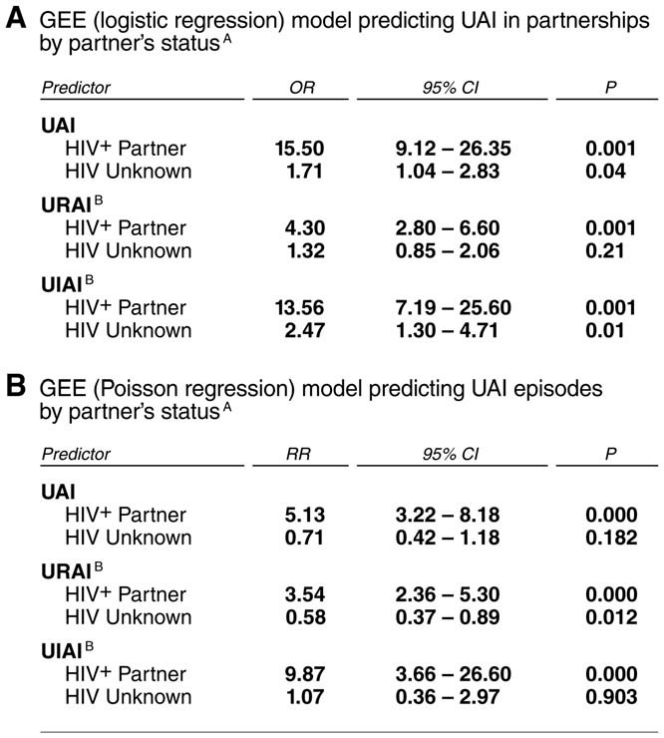
General estimating equation models predicting UAI in partnerships. The HIV-positive status of partner was a strong predictor of sexual practices in partnerships and for individual episodes of intercourse; partners of unknown status were not treated very differently from HIV-negative partners. The analysis also revealed strategic positioning by showing that positive participants were several times more likely to practice insertive rather than receptive UAI in serodiscordant partnerships. ^A^ HIV-negative partners are the reference group in all cases. ^B^ Designates the sexual position of the HIV-positive participant.

To investigate the link between partner infection status and partnership-specific practices, we used GEE logistic regression models. This analysis confirmed that once partners were selected, partner status was a strong predictor of sexual practices related to risk (Figure 3a). UAI was 15.5 times more likely to occur with a positive partner than a negative one (95% confidence interval [CI], 9.1–26.4). Receptive UAI was 4.3 times more likely in seroconcordant partnerships than with negative partners (95% CI, 2.8–6.6), but insertive UAI was 13.6 times more likely with positives (95% CI, 7.2–25.6). Thus, a pattern of strategic positioning occurred in cases of serodiscordant UAI, independent of individuals' typical preference for either insertive or receptive intercourse.

### Most Unprotected Intercourse Occurred in Seroconcordant Partnerships

We further elaborated this risk analysis with the frequency distribution of intercourse across partnerships. In exploratory analyses of the relationship between partnership-specific *acts of intercourse* and partner-status categories, partner status had an even greater effect on the distribution of intercourse across partnerships than on the distribution of partnerships. Of all unprotected intercourse acts in the past 3 months, 88.6% occurred in seroconcordant partnerships (Figure 2b.) Patterns of strategic positioning emerged with both negative and unknown-status partners, with episodes of insertive UAI accounting for only 2% and 6% of all unprotected insertive intercourse episodes, respectively.

GEE Poisson regression models showed that by the relative rates of UAI, receptive UAI, and insertive UAI were significantly higher in seroconcordant than in serodiscordant partnerships (Figure 3b).

### Failure to Consider Seroadaptive Behaviors Leads to Inflated Estimates of Epidemic Spread

**Figure 4:**
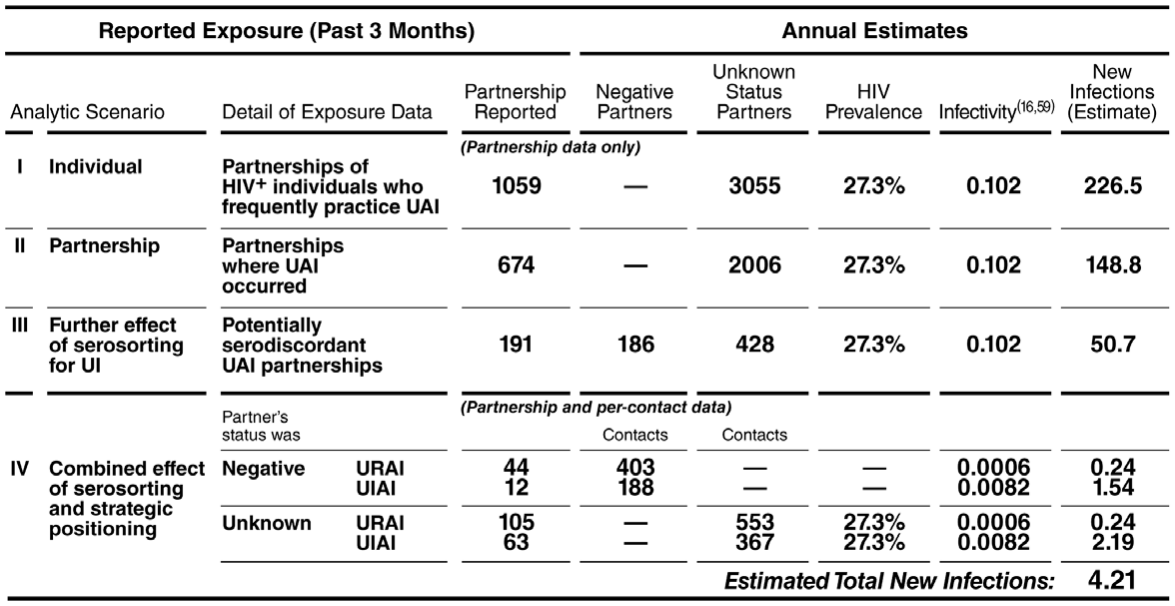
Estimates of potential new infections vary by analytic strategy and detail of data. Sexual behavior reported by this cohort could potentially contribute to as many as 227 infections in a year (I). Using the partnership rather than the individual as the unit of analysis and fully utilizing most of the exposure data, we estimate that as few as 4.2 new cases might occur (IV). The sexual choices of these HIV-positive individuals reduced potential new infections by 98.1%—a finding that would be missed in some analyses.

Estimates of epidemic spread of HIV have been calculated according to assumed average numbers of sexual partners and reported estimates of per-contact infectivity associated with various modes of contact [16], [58]. These estimates typically assume random selection of sexual partners and impose other conditions on mixing (e.g., that the most sexually active individuals would select partners also among the most active group). Incomplete information about partnerships and sexual behaviors leads to gross overestimation of the risk of HIV transmission in communities where seroadaptation was common, as in our sample (Figure 4). Based upon the sexual partnerships reported to us by 168 MSM, we estimate that they could contribute to as many as 227 new infections in a year, assuming random partnering if specific partnership information was not considered (Analytic Scenario 1: Individual). Considering only partnerships that included UAI rather than counting all individuals who reported any type of partnership, the estimate drops to 149 new infections (Analytic Scenario 2: Partnership). Considering the HIV status of partners with whom UAI occurred would reduce our estimate to 51 new infections (Analytic Scenario 3: Further effect of serosorting for UI). Taken together, these methodological and analytic considerations that allowed us to see serosorting and calculate its impact reduced our estimates of new infections to 22% of that from approaches that could not incorporate these choices.

Having collected data on partners' HIV status and specific sexual contacts within those partnerships, we estimated that this highly active and ostensibly risky group might contribute to as few as four new infections per year (Analytic Scenario 4: Combined effect of serosorting and strategic positioning). By including most of the detail regarding sexual choices, we estimated those choices potentially reduced new infections by 98%. Hence, failure to collect or consider seroadaptive tactics among seropositives could overestimate the risk of HIV transmission in these communities by more than 50-fold.

## Discussion

Our findings reveal extensive patterns of seroadaptation among HIV-positive MSM in San Francisco before 2005. In this sample, seroconcordant partnerships were preferentially selected by many individuals. However, the GEE analysis, which controlled for individual and partnership characteristics, confirmed the strong, independent association of sexual practices with partner status. Therefore, seroadaptation need not limit partner choices; rather, it informs sexual practices based on partner serostatus. Seroadaptation does not require or imply what has been called “viral apartheid.” Although 41.1% of men reported only seroconcordant partners in the past 3 months, many of these involved monogamous partnerships at that time. More than half of participants also selected partners who were of negative or unknown status. Also, while partners of unknown status were exposed to more risk than negatives, they were treated much more like negatives than positives, indicating that seroadaptation does not always require serodisclosure.

Although serosorting provided the opportunity to practice unprotected intercourse without risk of new infections, strategic positioning had clearly been adapted as a strategy to reduce the risk of transmission in serodiscordant partnerships or where the partner status was unknown. By our estimates, serosorting for unprotected intercourse, using condoms in potentially serodiscordant partnerships, and strategic positioning have considerable effects in reducing transmission of HIV-1 even among this ostensibly risky cohort.

The efficacy of strategic positioning has not been clinically evaluated, and it had not been proposed in HIV-prevention public health messages either in Australia where it was first identified or in San Francisco (up to or during this study period) [15]. However, convincing data on the relative risks of receptive and insertive UAI have long been available. For example, some of the earliest studies of behavioral risk factors for infection found a high association with receptive intercourse, while insertive intercourse posed no increased risk for infection [67]. This finding was confirmed by the earliest well-controlled study of HIV transmission in San Francisco [17]. Later studies of per-contact infectivity showed that receptive UAI with an infected partner was the riskiest sexual practice—more than 10-fold more likely to result in infection than insertive intercourse, which carried about as much risk as oral sex [16]. Information such as this was widely circulated in San Francisco. Clearly, strategic positioning had emerged alongside other seroadaptive tactics from the “ground up” and was, at the least, consistent with current scientific data on risk, if not officially informed by those findings.

We estimated that reported seroadaptive behaviors could reduce HIV transmissions from this group by 98% per year. Any new HIV infections are too many in our view. However, serosorting and strategic positioning profoundly reduced the potential number of estimated new infections. Thus, seroadaptation, especially among highly sexually active MSM, may be one of the most efficacious prevention strategies for HIV-positive MSM and one that arose from their own choices.

Although only a small fraction of prevention research has been designed to detect seroadaptation [68], evidence of seroadaptation appeared early in the epidemic and is found in diverse settings under the following conditions: (1) HIV testing must be common in the local risk group; (2) knowledge about risk factors for HIV transmission must be widespread; and (3) a culture of disclosure must exist wherein it is relatively safe or even routine to disclose HIV status to other members of the group.

We note that this constellation of conditions did not randomly appear, at least in the San Francisco gay community. High rates and frequencies of HIV voluntary counseling and testing occurred early in the epidemic and have been sustained for two decades by demand within the community. Yet, establishing accessibility and uptake of voluntary testing and counseling—the very first step in effective HIV prevention—remains a primary aim of HIV prevention today in communities around the world where demand has not already made it common practice. Proliferation of information about HIV transmission was accomplished in San Francisco by community-based organizations and other indigenous community infrastructures as much as by public health policy [69]. The culture of disclosure in San Francisco, reportedly manifest in 66% of the partnerships in this study, reveals an important feature of the community. Disclosure for HIV-positive individuals may be a tactic for accessing unprotected intercourse, but such disclosure can also make desired sexual activities less likely, highlighting that disclosure is driven by an element of altruism. Disclosure for negative individuals is also based, in part, on a common understanding that partners will have concern for their health. Gay communities in San Francisco and elsewhere provide a context that is particularly ripe for fostering seroadaptive tactics to the extent that sexual conduct is oriented by regard for the health of the other. Such sexual conduct is supported by community organization and structure.

Although it is difficult to test the reliability and validity of the measures used in this study, other available reports support our findings and suggest that seroadaptive tactics in addition to condoms or abstinence emerged early in the epidemic under these conditions in several locales internationally [14], [19]–[21], [23].

In the presence of seroadaptive strategy, studies of HIV risk are no longer informative when based only on individual characteristics, unprotected intercourse, and number of partners. Sexual network data and analytic strategies based on partnerships as the unit of analysis are well suited to understanding current risk reduction tactics and informing epidemiological modeling. The strong pattern of seroadaptative tactics reported here suggests that STI surveillance data were uninformative as a surrogate indicator of trends in HIV incidence. Indeed, since this seroadaptive strategy was focused on HIV and not bacterial STIs, increasing rates of other STIs may indicate a growing and successful seroadaptive culture that could decrease HIV transmission while unprotected intercourse becomes more common.

We found that collecting egocentric sexual network data was not expensive and involved an acceptable burden to participants. Furthermore, a questionnaire designed to capture a more complete range of sexual experiences, encounters, and choices was far more acceptable to participants than approaches designed to focus on seroconversion risk and pathological covariates that overlook tactics that participants have learned and implemented to prevent transmission. Indeed, respondents frequently enjoyed reporting specific characteristics about specific partnerships. Describing activities with individual friends and lovers is more engaging than answering abstract questions about numbers of partners and categories of acts; such questions are often alien to the respondents' way of thought. For example, the question “With how many partners have you had intercourse without a condom?” involves consideration of many experiences with several people in a mental calculation that many respondents find complicated and threatening. The egocentric data about each partnership are easier for the respondent to provide and yield detailed datasets that are readily analyzed with recently developed GEE models that control for participant and partnership characteristics. This information also lends itself to complex diagramming and mathematical modeling. Consideration of assortative (and disassortative) partnering can be informed with additional information about the age and ethnicity of the partner. Information about concurrence of sexual partnerships has likewise proven a singularly powerful tool for modeling epidemic spread [49], [54], [70], [71]. All of these cofactors useful in epidemiologic studies of HIV and evaluations of HIV prevention programs can easily be collected within the framework of sexual network methodology.

Sorting out seroadaptation with sexual network data is now a straightforward and manageable process, made essential by harm reduction initiatives of those living with HIV-1. Methods used to study groups at risk for HIV-1 infection must be at least as sophisticated as the social adaptations evolving in those groups.

### Limitations

This study is limited by the sampling frame, which included only seropositive men in the San Francisco area, most of whom had been diagnosed many years before. Men who responded to and enrolled in a study of HIV superinfection may have been motivated to do so precisely because they perceived that their preference for these seroadaptive behaviors put them at increased risk for superinfection. Thus the strong patterns of seroadaptation described here may be biased by self selection of avid serosorters into the study. Further, seropositive serosorting may be simpler than seronegative serosorting, which is complicated by concerns that the putative seronegative partner may have become acutely infected since the last seronegative test.
